# Microfluidic Electrospinning Core–Shell Nanofibers for Anti‐Corrosion Coatings With Efficient Self‐Healing Properties

**DOI:** 10.1002/advs.202409751

**Published:** 2024-12-18

**Authors:** Qingqing Tang, Cuiping Ji, Guoying Wei, Jing Hu, Feifan Chang, Benfeng Zhu, Li Ren, Dongliang Peng

**Affiliations:** ^1^ College of Materials and Chemistry China Jiliang University Hangzhou 310018 P. R. China; ^2^ Department of Materials Science and Engineering College of Materials Xiamen University Xiamen 361005 P. R. China

**Keywords:** anti‐corrosion, composite coatings, core–shell nanofibers, microfluidic electrospinning, self‐healing

## Abstract

Self‐healing materials have been extensively explored in metal anti‐corrosion fields. However, improving the self‐healing efficiency remains a significant work that severely limits their further development. Here, a strategy to fabricate anti‐corrosion coatings with efficient self‐healing properties based on microfluidic electrospinning technologies and UV‐curable healing agents is reported. The damaged composite coating contains core–shell nanofibers that can be completely healed within only 30 min, indicating an outstanding healing efficiency. The corrosion current density (*I_corr_*) of the composite coatings containing core–shell nanofibers (abbreviated as composite coatings) is lower than the coatings without any fibers (abbreviated as pure resin coatings) during the test of repeated damage and healing cycles, showing superior resistance to corrosion and repeated self‐healing property. The composite coating has even better mechanical properties such as tensile strength, bending strength, and impact strength than the pure resin coating, which are explained by simulating the deformation process. These excellent properties greatly improve the practicability of self‐healing coatings in the application of anti‐corrosion, especially in some special fields.

## Graphical abstract

Here, a strategy to fabricate anti‐corrosion coatings with efficient self‐healing properties based on microfluidic electrospinning technologies and UV‐curable healing agents is reported. The damaged composite coating contains core–shell nanofibers that can be completely healed within only 30 min, indicating an outstanding healing efficiency. The healed composite coating can protect the aluminum substrate from corrosion, showing superior anti‐corrosion properties.

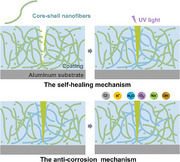

## 1 Introduction

Metals and alloys are everywhere in our daily lives, but most inevitably suffer from corrosion in working environments, which results in major financial losses, hazardous environmental damage, or even deadly accidents in a variety of industries, from chemical engineering to pipelines, ships, and highways.^[^ ^1^, ^2^ ^]^ Thus, numerous anti‐corrosion strategies have been developed to improve the resistance to corrosion of metallic materials. With the growth of surface engineering techniques, several techniques have emerged, such as electrodeposition,^[^ ^3^, ^4^ ^]^ electroless plating,^[^ ^5^ ^]^ and covering with an organic coating.^[^ ^6^, ^7^, ^8^ ^]^ Among them, the organic coating can not only protect the substrate from common corrosion but also endow the system with other functions such as superhydrophobic,^[^ ^9^ ^]^ anti‐icing,^[^ ^10^ ^]^ anti‐fogging,^[^ ^11^, ^12^ ^]^ etc. However, microcracks usually appear when coatings are damaged by certain factors such as impact or scratches, resulting in the corrosion of the substrate and the shortened service life.^[^ ^13^, ^14^, ^15^, ^16^ ^]^ Then, self‐healing coatings, whose cracks can be healed or other properties such as hydrophobicity and hydrophilicity,^[^ ^17^ ^]^ antibacterial activity,^[^ ^18^ ^]^ and corrosion resistance^[^ ^19^ ^]^ can be recovered, have been widely exploited.

Thereinto, external self‐healing coatings, whose healing property relies on the encapsulated healing agents in containers (including microcapsules and microchannels) to avoid premature reactions, have been widely used in anti‐corrosion applications.^[^ ^20^ ^]^ Among them, microcapsules have been more widely studied due to their relatively mature manufacturing technology and the wide variety of materials available for the core and the shell. For example, Chong et al.^[^ ^21^ ^]^ reported the coating with anti‐bacterial and corrosion protection performances in seawater, consisting of multifunctional microcapsules prepared by in situ polymerization. Feng et al.^[^ ^22^ ^]^ reported the anti‐corrosion and self‐healing coating containing microcapsules prepared by interfacial polymerization. Nevertheless, the self‐healing process of microcapsules‐based coatings usually takes a long time due to the limited number of microcapsules in the damaged region. The aggregation of microcapsules often leads to uneven distribution of healing agents in coatings and decreases mechanical properties of coatings, making it difficult to ensure the reliability of self‐healing properties. The fabrication process of microcapsules is usually complex and time‐consuming. These issues limit the further development of microcapsules in self‐healing coatings.

Microchannel systems, mainly fibers, have attracted much attention in self‐healing and anti‐corrosion coatings because of the uniform size of fibers, efficient preparation process, and usually uniform distribution in the matrix. The common fabrication methods for nanofibers include microfluidic spinning,^[^ ^23^ ^]^ electrospinning,^[^ ^24^ ^]^ microfluidic electrospinning,^[^ ^25^, ^26^ ^]^ melt spinning,^[^ ^27^ ^]^ solution blowing spinning,^[^ ^28^ ^]^ and so on. For example. Ji et al.^[^ ^29^ ^]^ reported a coating on the steel based on the core–shell fibers with self‐healing and pH‐responsive properties through the electrospinning technology. Cao et al.^[^ ^30^, ^31^ ^]^ reported a coating with the core–shell nanofiber network, which showed corrosion‐diagnosing and anti‐corrosion properties for the carbon steel substrate. However, it is still a big challenge for both the microcapsule‐ and microchannel‐based coatings to achieve self‐healing properties with high efficiency, which is desirable and urgent in practical applications.

In recent years, microfluidic electrospinning technology has gradually attracted attention due to its advantages such as precisely controlling the multi‐phase flow to regulate the structure of fibers by microfluidic technology, and fast preparation of fibers without high temperature as well.^[^ ^32^ ^]^ Herein, we developed an anti‐corrosion coating with highly efficient self‐healing properties based on microfluidic electrospinning nanofibers and UV‐curable healing agents. Nano‐scale core–shell nanofibers with controllable and uniform size were prepared by the microfluidic electrospinning technology benefiting from the precise control system of the microfluidic system and efficient electrospinning system. The microcracks of composite coatings containing core–shell nanofibers (abbreviated as composite coatings) can be completely healed within 30 min due to the encapsulation of UV‐curable healing agents in nanofibers and the uniform distribution of nanofibers in coatings. The self‐healed composite coatings show effective anti‐corrosion properties. Besides, polyacrylonitrile (PAN) is selected as the shell material due to its excellent ductility, and core–shell nanofibers endow coatings with better mechanical properties, for instance, tensile strength, bending strength, and impact toughness, which will promote the practicality of self‐healing coatings for the prevention of metal corrosion.

## 2 Results and Discussion

### 2.1 Fabrication and Characterizations of Nanofibers

**Figure 1:**
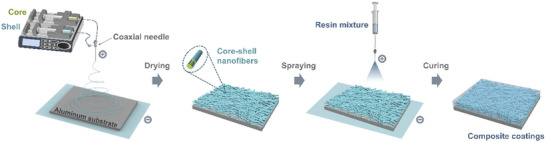
Schematic illustration of the fabrication for core–shell nanofibers and composite coatings.

**Figure** **1** schematically illustrates the fabrication of composite coatings including the microfluidic electrospinning process and the resin mixture deposition process. Briefly, core–shell nanofibers were prepared by mixing solutions in the microfluidic chip (coaxial needle) and regulating the structure by the microfluidic control system. The outer needle was attached to syringe pump 1 filled with shell materials (PAN solution) and the inner one was attached to syringe pump 2 filled with core materials (healing agents), respectively. Then, core–shell nanofibers were collected on an aluminum substrate under the high‐voltage electric field. After drying, the resin mixture was sprayed on the aluminum substrate covered with nanofibers under the high‐voltage electric field until the nanofibers were completely covered. Finally, composite coatings were obtained after curing.

**Figure 2:**
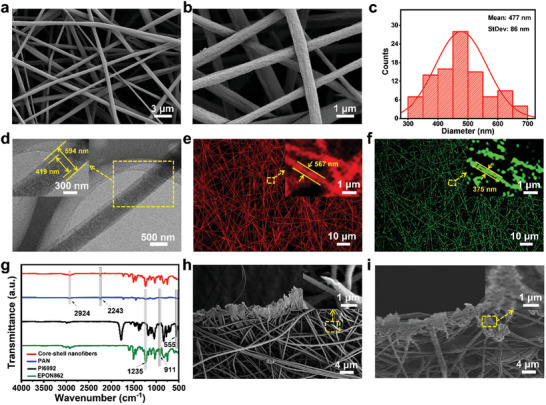
a, b) Scanning electron microscopy (SEM) images of core–shell nanofibers. c) The diameter distribution histogram. d) The transmission electron microscopy (TEM) image of the core–shell nanofiber. CLSM images of nanofibers with e) rhodamine B in the shell and f) fluorescein in the core. g) Fourier transform infrared (FTIR) spectra of core–shell nanofibers, PAN, triarylsulfonium hexafluorophosphate salts (PI 6992), and Bisphenol F epoxy resin (EPON862). Cross‐section SEM images of cut h) pure solid PAN nanofibers and i) core–shell nanofibers.

Core–shell nanofibers are continuous without breakage, and uniform without any bead strings (**Figure** **2**a,b). And the average diameter is 477 nm obtained by the software Image J 100 times (Figure 2c). Figure 2d displays a nanofiber with a diameter of 594 nm and a core diameter of 419 nm, the boundary between the core and the shell is clear, indicating a core–shell structure of the nanofiber. The volume ratio of core materials is calculated to be ≈35%. Confocal laser scanning microscopy (CLSM) was used to visualize the core and shell of nanofibers. Specifically, each fluorescent dye has its own specific emission wavelength, displaying different colors at their respective excitation wavelengths. Emission peaks of rhodamine B (red curve) and fluorescein (green curve) are 581 and 515 nm, respectively (Figure S1, Supporting Information), displayed as red and green. The fluorescent material rhodamine B was added to the shell, and the other fluorescein was added to the core. CLSM images show that the size of the fiber shell (567 nm) represented in red is larger than that of the core (375 nm) of the same fiber represented in green (Figure 2e,f), suggesting the core is encapsulated in the shell, confirming the core–shell nanostructure.

Figure 2g shows FTIR spectra of core–shell nanofibers (red curve), PAN (blue curve), the EPON862 (green curve), and the PI 6992 (black curve), respectively. The characteristic peak of PAN at 2243 cm^−1^ determines the vibration of the C≡N bond, and the distinctive peak of the ‐CH_2_ group appears at 2924 cm^−1^. For core material EPON862, absorption peaks at 1235 and 911 cm^−1^ were ascribed to the C─O─C bond and the epoxy group, respectively. The peak at 555 cm^−1^ originated from PI 6992 indicating the presence of the P─F bond. The spectra of the core–shell nanofiber showed all of the characteristic peaks of the shell and core materials, indicating the encapsulation of core agents successfully. The fact that functional groups are retained shows there is no chemical reaction between the shell and core materials, rather, it is a physical process when the encapsulation of core materials.

When damaged, the edges of pure PAN solid nanofibers are clear (Figure 2h), while there are accumulated healing agents at the edge of core–shell nanofibers (Figure 2i), indicating that healing agents can flow out smoothly, thus further ensuring the reliability of self‐healing property.

### 2.2 Fabrication and Characterization of Coatings

The nanofiber layer is loose and porous (Figure 2i), which is susceptible to external damage such as scratching, pressing, and impact, and it is difficult to achieve corrosion resistance. Hence, the epoxy resin mixture was used to cover nanofibers, and then the composite coating was obtained after curing. The thickness of the composite coating is ≈46 µm (Figure S2a, Supporting Information). All elements, especially the F, P, and S elements derived from core–shell nanofibers, are uniformly distributed (Figure S2b–g, Supporting Information), indicating that nanofibers are uniformly distributed in the composite coating.

The adhesion to the aluminum substrate of the composite coating was assessed by the tape‐peeling test, the results are shown in Figure S2 h (Supporting Information). According to the cross‐cut test in ISO‐2409 standard,^[^ ^33^ ^]^ the composite coating was cross‐cut into a checkerboard‐like lattice, and then peeled off twice with a tape (Video S1, Supporting Information), as shown in Figure S2 h (Supporting Information), edges of the cuts are quite smooth, and there is no peeling from any squares in the lattice, which meets the requirements of surface appearance class 0 (ISO‐2409). The result suggests that the excellent adhesion between the composite coating and the aluminum substrate, and the coating is potential in anti‐corrosion application effectively.

### 2.3 Self‐Healing Properties

**Figure 3:**
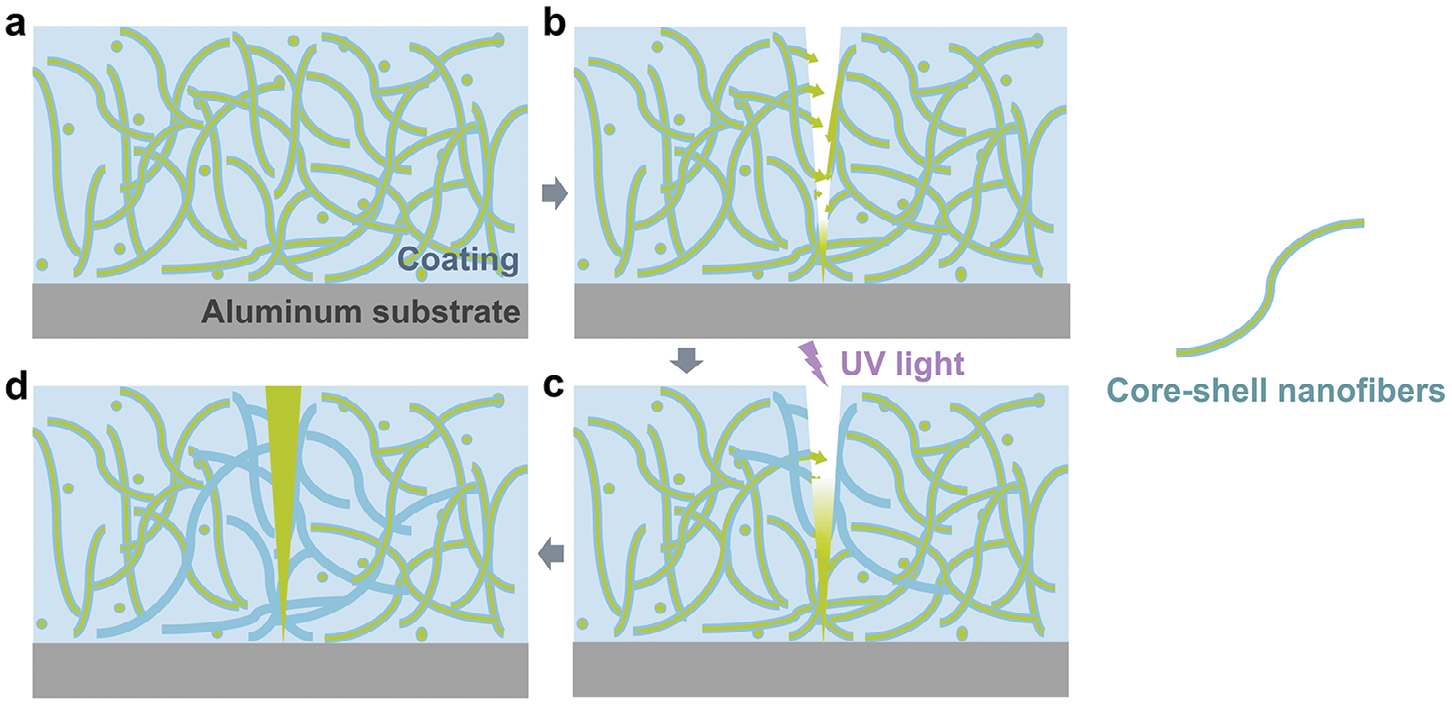
Self‐healing mechanism of composite coatings. a) The original composite coating. b) The damaged composite coating with cracks. c) The cracked coating under UV radiation. d) The healed coating.

**Figure** **3** shows the self‐healing mechanism of composite coatings. When a crack appears in the coating, core–shell nanofibers break, and healing agents pour out of the core and fill the crack. Then, the coating is radiated under UV light to polymerize healing agents. Finally, the coating is healed, that is, the damage and healing cycle is completed.

**Figure 4:**
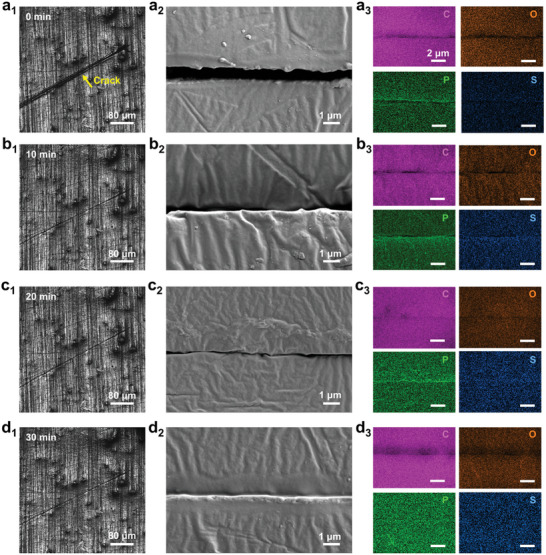
OM, SEM images, and corresponding energy dispersive spectroscopy (EDS) maps of the composite coating with a crack a) before and after UV radiation for b) 10 min, c) 20 min, and d) 30 min.

**Figure** **4** shows optical microscopy (OM) and SEM images of a crack in the composite coating before and after UV radiation for 10, 20, and 30 min, respectively. Here, UV lamps with a wavelength of 310 nm were used for irradiation according to the UV absorption spectrum of PI 6992 (Figure S3, Supporting Information). In detail, Figure 4a shows a visible crack in the coating because of damage, and elements P and S unique to PI 6992 were detected in the crack, indicating that the healing agent can flow smoothly from nanofibers and quickly fill the crack. With the increase in UV radiation time, the crack was gradually repaired due to the polymerization of healing agents. After UV radiation for 10 min, the crack narrowed (Figure 4b), and the coating was almost healed after 20 min of UV radiation (Figure 4c). After only 30 min of UV radiation, the coating was completely healed (Figure 4d), indicating that the healing effectiveness of the composite coating is excellent, which results from the successful encapsulation of UV‐curable healing agents with good flowability in core–shell nanofibers.

Compared with the coating composited with core–shell nanofibers, there is no change in the crack of coatings without any fibers (abbreviated as pure resin coatings) and composite coatings containing pure PAN solid nanofibers (abbreviated as PAN@coatings) before and after UV radiation for 30 min (Figure S4, Supporting Information), that is, the two has no self‐healing properties. This confirms that the encapsulated healing agents contribute to the self‐healing properties.

### 2.4 Anti‐Corrosion Properties

**Figure 5:**
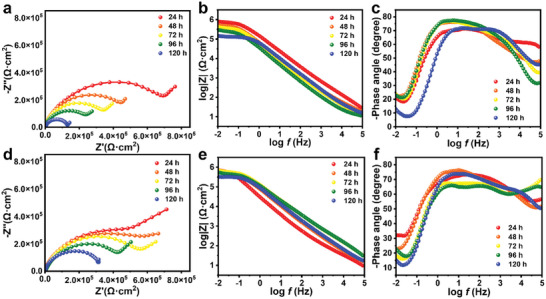
Nyquist and Bode plots of a–c) the intact pure resin coating and d–f) the intact composite coating during the immersion process.

Electrochemical impedance spectroscopy (EIS) measurements were conducted to confirm the impact of core–shell nanofibers on the long‐term durability of coatings. **Figure** **5** shows the Nyquist spectra, the Bode magnitude, and phase angle plots of aluminum substrates with different coatings immersed in a 5 wt.% NaCl solution at different times. The Nyquist curves of coatings are shown in Figure 5a, d, where the radius of the arc and the speed of charge transfer are highly correlated.^[^ ^34^ ^]^ Overall, the larger the radius, the coating has the better corrosion resistance. During the immersion process, the radius of the composite coating is larger than the pure resin coating, confirming the enhanced charge transfer resistance of the composite coating, i.e., better corrosion resistance. Besides, the low‐frequency impedance (|Z|_0.01_ _Hz_) is a widely recognized semi‐quantitative parameter to evaluate the corrosion resistance of coatings. For pure resin coatings (Figure 5b), |Z|_0.01_ _Hz_ values decreased from 8.182 × 10^5^ Ω·cm^2^ (24 h) to 1.467 × 10^5^ Ω·cm^2^ (120 h). As the immersion time increases, the |Z|_0.01_ _Hz_ gradually decreases, indicating that the anti‐corrosion property of pure resin coating decreased, because the corrosive medium gradually penetrates, weakening the corrosion resistance of the coating. Compared to the pure resin coating, the |Z|_0.01_ _Hz_ value of the composite coating was 8.433 × 10^5^ Ω·cm^2^ after being immersed in 5 wt.% NaCl solution for 24 h, which was higher than that of pure resin coating, indicating the core–shell nanofibers provided an extended diffusion path and increased the diffusion interface of corrosive ions, the corrosion rate has slowed down. After immersion for 120 h, due to the continuous infiltration of corrosive media, the value of |Z|_0.01_ _Hz_ decreased to 3.263 × 10^5^ Ω·cm^2^, which was still higher than the pure resin coating, indicating that the addition of core–shell nanofibers significantly improves the protective performance of the coating, further demonstrating its advantages of long‐term protection.

**Figure 6:**
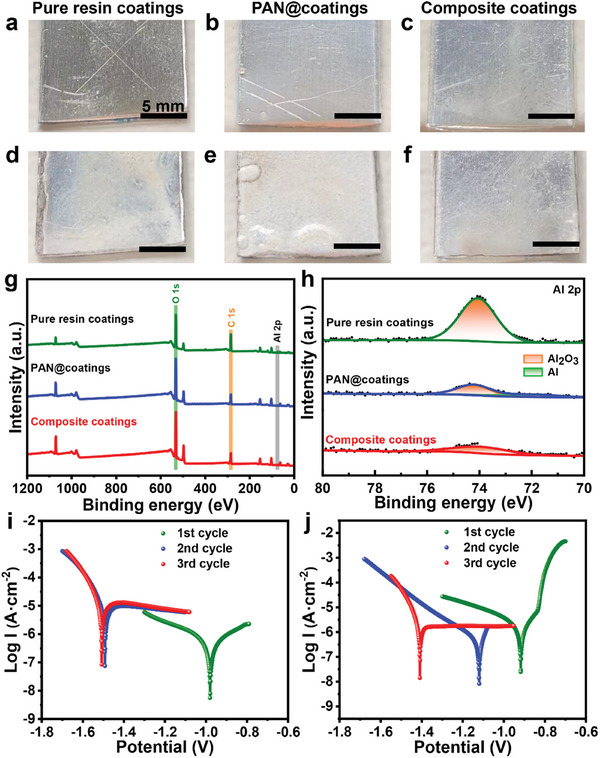
Digital photographs of three coatings a–c) before and d–f) after immersed in 5 wt.% NaCl solution for 48 h. g) XPS spectra of pure resin coatings, PAN@coatings, and composite coatings in cracked sites. h) The high‐resolution single peak of Al 2p. Potentiodynamic polarization curves of i) pure resin coatings and j) composite coatings under different damage and healing cycles.

The salt water immersion experiment was carried out to intuitively evaluate the anti‐corrosion property of coatings. After the damaged coatings radiated under UV conditions, there are still noticeable cracks on the surface of the pure resin coating (**Figure** **6**a) and the PAN@coating (Figure 6b), while cracks on the surface of the composite coating (Figure 6c) almost disappear. After that, three coatings were immersed in 5 wt.% NaCl solution at ambient temperature for 48 h. Figure 6d,e show the accumulation of many white corrosion products on the surface of the PAN@coating and pure resin coating, respectively, especially around the cracks. There is less white product on the surface of the composite coating than the two coatings (Figure 6f), especially around the cracks. X‐ray photoelectron spectroscopy (XPS) spectra are employed to characterize the composition and the content of white corrosion products, the survey spectrum and the Al 2p spectrum of white corrosion products in cracked sites are shown in Figure 6g,h. The valence state of Al changes before and after corrosion, i.e., the metal aluminum (Al^0^) and Al^3+^, which belong to the corrosion product Al_2_O_3_. Thus, the concentration of Al 2p atom is employed to evaluate the anti‐corrosion property of coatings and the fitting results are summarized in Table S1 (Supporting Information). The Al 2p concentration of the pure resin coating is up to 9.33%, and that of the PAN@coating is 4.79%, while that of the composite coating is 3.26%, almost one‐third of the former, indicating the least amount of corrosion products, further confirming that the composite coatings have active protection properties.

The potentiodynamic polarization experiment was conducted to characterize the anti‐corrosion properties of the composite coating after each healing cycle (Figure 6i,j). The polarization curve‐derived parameters include corrosion potential (*E_corr_*) and corrosion current density (*I_corr_*) are shown in **Table** **1**. The *I_corr_* is corresponded to the dynamic corrosion rate,^[^ ^35^, ^36^ ^]^ the smaller the *I_corr_*, the better the anti‐corrosion property. After the first damage and healing cycle, the *I_corr_* value of composite coatings was smaller than pure resin coatings, showing better anti‐corrosion properties due to its self‐healing properties. For pure resin coatings, the *I_corr_* value was increased from 7.217 × 10^−7^ A·cm^−2^ (1st cycle) to 1.847 × 10^−5^ A·cm^−2^ (3rd cycle), increased by two orders of magnitude, indicating that the pure resin coating had lost its protection for the aluminum substrate. Compared to the pure resin coatings, the composite coating *I_corr_* value was increased by an order of magnitude from 1.573 × 10^−7^ A·cm^−2^ to 3.663 × 10^−6^ A·cm^−2^ after the third cycle. The *I_corr_* of the composite coating is always lower than that of the pure resin coating due to the reliable self‐healing properties, indicating that the composite coatings have good durability.

**Table 1:** The polarization curve‐derived parameters of pure resin coatings and composite coatings under different damage and healing cycles.

Samples	Healing cycle	*E_corr_* [V]	*I_corr_* [A·cm^−2^]
Pure resin coatings	1st cycle	−0.980	7.217 × 10^−7^
	2nd cycle	−1.492	1.476 × 10^−5^
	3rd cycle	−1.508	1.847 × 10^−5^
Composite coatings	1st cycle	−1.120	1.573 × 10^−7^
	2nd cycle	−0.918	4.636 × 10^−7^
	3rd cycle	−1.409	3.663 × 10^−6^

Furthermore, the copper sulfate solution pitting test is employed to verify the anti‐corrosion properties of different coatings. After the three damaged coatings radiated under UV conditions, the prepared copper sulfate solution was dropped on their surface. As shown in Figure S5 and Video S2 (Supporting Information), the corrosion process was visible in cracked sites of the pure resin coating and the PAN@coating after 30 s of dripping, while there was no change in the composite coating. By 300 s, the two coatings had been completely corroded, while the composite coating was still only partially corroded, which further confirms the outstanding anti‐corrosion and self‐healing properties of composite coatings. The video was played at a speed of 4×.

**Figure 7:**
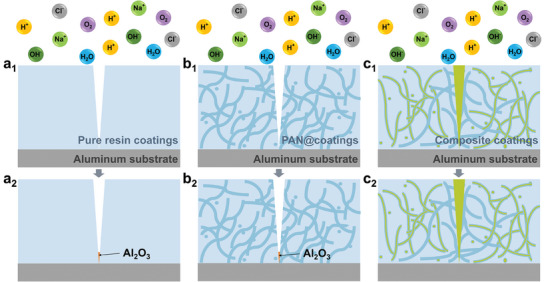
Corrosion mechanism of a) pure resin coatings and b) PAN@coatings. c) Anti‐corrosion mechanism of composite coatings.

**Figure** **7** schematically illustrates the corrosion mechanism of the pure resin coating and the PAN@coating, and the anti‐corrosion mechanism of the composite coating. Once coatings are damaged, aluminum substrates covered with pure epoxy coating (Figure 7a) and the PAN@coating (Figure 7b) will be directly exposed to an external medium because there is no additional protection available. Then, corrosion will occur at the exposed area due to the reaction of Al with corrosion media including H_2_O, O_2_, and Cl^−^, resulting in the valence state of Al changed and the accumulation of Al_2_O_3_, proving by the XPS spectra (Figure 6g,h). As for composite coatings (Figure 7c), healing agents will flow out from nanofibers due to the damage and fill the crack quickly, thus avoiding the contact of the aluminum substrate with the external medium in time. After curing, the healed coating can protect the aluminum substrate from corrosion, which can be called active corrosion protection.

### 2.5 Mechanical Properties

**Figure 8:**
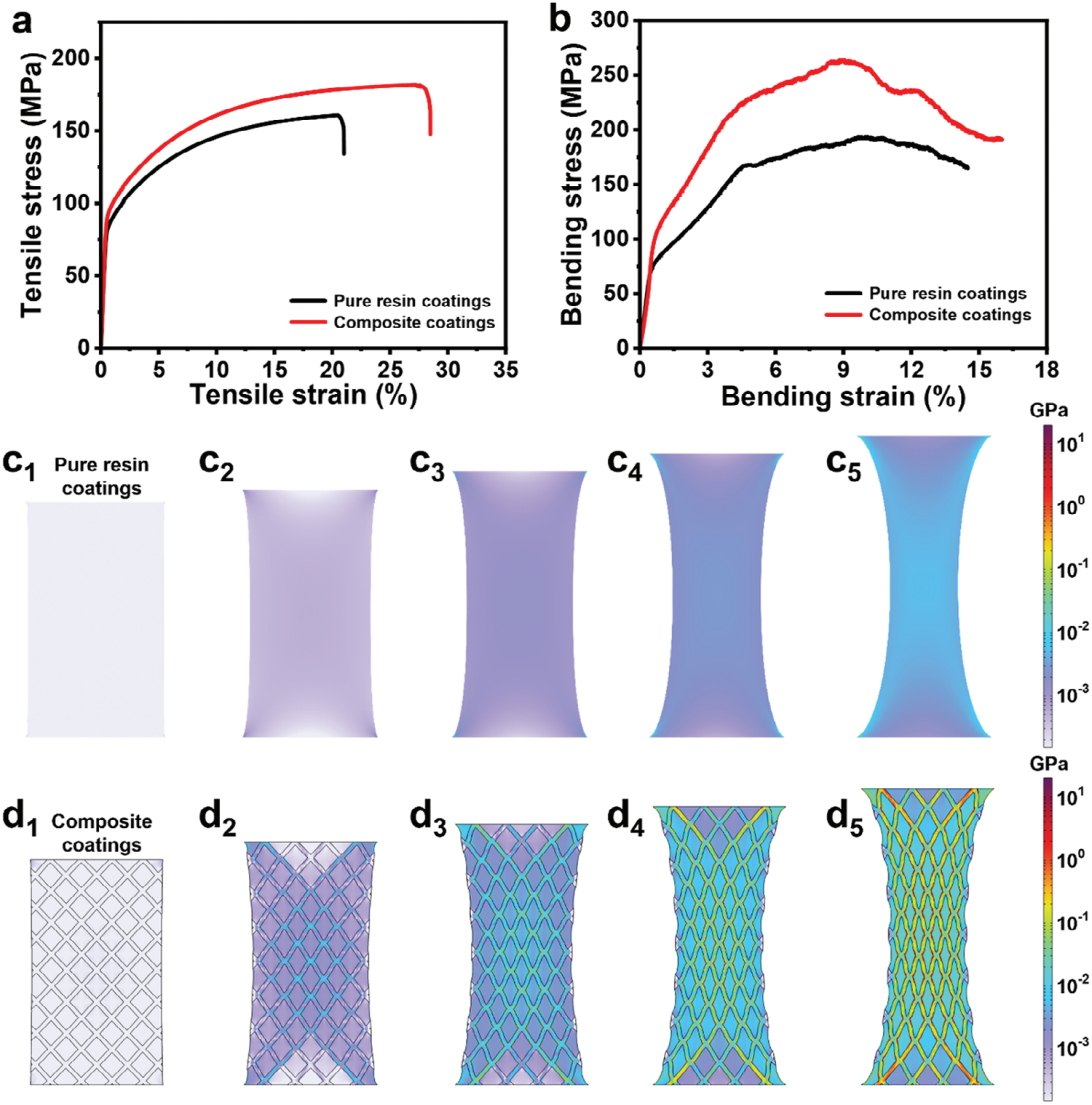
a) Tensile stress–strain and b) bending stress–strain curves of coatings. c‐d) Models of coatings and schematic diagrams of the simulation of the deformation of coatings during stretching.

The tensile experiment and three‐point bending test were carried out to assess the mechanical properties of coatings, tensile stress–strain curves, and bending stress–strain curves of pure resin coatings and composite coatings are shown in **Figure** **8**a, b, details are listed in Table S2 (Supporting Information). The tensile strength of the pure resin coating is 162.83 MPa, while that of the composite coating is 182.90 MPa, indicating an increase of ≈11%. The elongation at break of the pure resin coating is 23.94%, while that of the composite coating is 29.61%, indicating an increase of ≈19%. In addition, the larger the area enclosed by the curve and the horizontal axis, the larger the toughness. Thus, the toughness of composite coatings is much larger than pure resin coatings. The bending strength of the pure resin coating is 196.95 MPa, while that of the composite coating is 250.97 MPa, indicating an increase of ≈22%, which is related to the improvement in flexibility, further confirming that composite coatings have superior mechanical properties. Besides, a charpy pendulum impact test was carried out to verify the impact toughness of coatings,^[^ ^37^ ^]^ and results are shown in Figure S6 and Table S2 (Supporting Information). Among them, the impact toughness of the pure resin coating is 15.93 J·cm^−2^, while that of the composite coating is 16.77 J·cm^−2^, indicating an increase of ≈5%. These results suggest that doped core–shell nanofibers not only do not reduce mechanical properties of resins as usual as dopants such as microcapsules but also interestingly improve mechanical properties of coatings. This may be ascribed to the uniform distribution and good compatibility of core–shell nanofibers in the multifunctional coating, which allows the stress to be evenly transferred and distributed within the composite coating, and core–shell nanofibers made of materials different from resins may also help to absorb more energy. In summary, core–shell nanofibers can not only impart self‐healing and anti‐corrosion properties to coatings but also improve the mechanical properties of coatings.

Two finite element models were constructed to simulate the deformation of coatings during stretching (Figure 8c,d). When both ends of the coating are stretched simultaneously, compared to the pure resin coating, the stress in the composite coating is mainly transmitted along the nanofibers and concentrated on the fibers. Due to the uniform distribution of nanofibers, a dense network is formed in the composite coating, and nanofibers are in contact with each other and tangled, like hooks and loops in velcro tape,^[^ ^38^, ^39^, ^40^ ^]^ creating a bridging effect that effectively reduces the stress concentration inside the coating. Besides, the PAN material as a shell has good ductility and also endows nanofibers with good flexibility. As a result, the composite coating can resist or withstand greater external forces, i.e., has a better mechanical property.

## 3 Conclusion

In summary, we have demonstrated an anti‐corrosion coating with highly effective self‐healing properties based on microfluidic electrospinning technology. The damaged multifunctional composite coating can be completely healed within 30 min due to the encapsulation of UV‐curable healing agents with good flowability. Compared with pure resin coatings without any nanofibers and composite coatings containing solid nanofibers, composite coatings containing core–shell nanofibers have better anti‐corrosion properties. Besides, compared to the pure resin coating, the composite coating shows superior mechanical properties in terms of tensile strength, bending strength, and impact toughness due to the stress dispersion caused by nanofibers. These excellent properties make the coating more practical and promising in terms of high‐level metal corrosion protection.

## 4 Experimental Section

### Materials

PAN (Mw = 150 kDa), glycidyl 2‐methylphenyl ether (technical grade, 90%), triarylsulfonium hexafluorophosphate salts (PI 6992, 50% in propylene carbonate), and rhodamine B (AR, 90%) were purchased from Shanghai Macklin Biochemical Technology Co., Ltd. The solvent N, N dimethylformamide (DMF, ≥99.5%), and fluorescein (AR, 90%) were purchased from Shanghai Aladdin Biochemical Technology Co., Ltd. The Bisphenol F epoxy resin (EPON862) was supplied from Hexion Co., Ltd. Polyamide (PA) was purchased from Tianjin Haiyan Chemical Co., Ltd. Hydrochloric acid (HCl), copper sulfate (CuSO_4_) and sodium chloride (NaCl) were purchased from Hangzhou Gaojing Fine Chemical Co., Ltd. All reagents were directly used in the experimental process without further purification.

### Characterization

Energy dispersive spectroscopy (EDS) maps of coatings, and morphologies of core–shell nanofibers and coatings were characterized with scanning electron microscopy (SEM, Sigma 300, ZEISS) after gold sputtering. Emission spectra of fluorescent agents were characterized with a fluorescence spectrometer (FL‐3, HORIBA). The core–shell nanostructure was characterized with transmission electron microscopy (TEM, JEM‐F200, JEOL) and confocal laser scanning microscopy (CLSM, FV1000, Olympus). Fourier transform infrared (FTIR) spectra were obtained from the FTIR spectrometer (Nicolet iS20, Thermo). UV–vis absorption spectrum of the cationic photoinitiator PI 6992 was obtained with a Shimadzu UV‐3600i Plus spectrophotometer at room temperature in the wavelength range of 200–600 nm.

To evaluate the self‐healing property of composite coatings, the composite coating was artificially scratched by a blade, and then the damaged area radiated under UV conditions (310 nm, 114 mW·cm^−2^) for 10, 20 and 30 min, respectively. The self‐healing process was observed through SEM and optical microscopy (OM, GP‐200MRT).

The corrosion behavior of coatings was measured with an electrochemical workstation (CHI660E, CH Instruments). Tests were performed by a standard three‐electrode system: the working electrode was a coated aluminum substrate, and platinum foil was the counter electrode, saturated calomel electrode (SCE) was the reference electrode. Additionally, 3.5 wt.% NaCl solution was used as the electrolyte solution and the exposed area of the working electrode was 1 cm^2^. Before testing, coatings were kept at open circuit potential (OCP) for 30 min. The corrosion potential (*E_corr_*) and corrosion current density (*I_corr_*) were derived from the potentiodynamic polarization experiment at a scan rate of 0.01 V·s^−1^.^[^ ^33^ ^]^ The electrochemical impedance spectroscopy (EIS) was measured at ambient temperature equally, and the frequency was scanned in the range from 10^−2^ to 10^5^ Hz. Damaged coatings after radiation under UV conditions were immersed in 5 wt.% NaCl solution for 48 h, and dried at 50 °C for 2 h to remove superfluous moisture. Corrosion products at cracked sites were characterized with X‐ray photoelectron spectroscopy (XPS, K‐Alpha, Thermo) with a microfocus monochromatic Al Kα X‐ray source (1486.6 eV), the strongest hydrocarbon (C 1s) at 284.8 eV was calibrated. All spectra were deconvoluted with Avantage software. A copper sulfate pitting test and wet corrosion test were carried out to supplement the corrosion resistance of the healed composite coating. The copper sulfate solution (41 g·L^−1^ CuSO_4_、35 g·L^−1^ NaCl and 0.1 mol·L^−1^ HCl)was dropped directly onto the surface of the coatings.

The mechanical properties of coatings (75 × 15 mm) were measured using an electronic universal testing machine (INSTRON 5982, Instron), and the tensile experiment was conducted with both ends clamped at the stretching rate of 5 mm·min^−1^,^[^ ^41^ ^]^ the three‐point bending experiment was conducted at a loading rate of 2 mm·min^−1^, and the average value was obtained from three samples. The finite element simulation software COMSOL6.0 was used to simulate the stress distribution in the composite coating during the tensile process. A charpy pendulum impact test was performed on an impact tester (ZBC3302‐A, MTS) according to the GB/T 229–2020 standard of China. The tape‐peeling test was performed on a cross‐cut tester (QFH‐A, Aipli). In order to verify test reliability and reproducibility, for each group, three specimens were evaluated in order for the average.

### Preparation of Core–Shell Nanofibers

The core–shell nanofiber with healing agents encapsulated was produced by the electro‐microfluidic spinning instrument (JNS‐MS‐05‐ME02, J@NUS), including the coaxial needle as the microfluidic chip and the microfluidic control system. Parameters for the microfluidic electrospinning process were listed as follows. The coaxial needle was a 22/17 (inner/outer) gauge needle, and the diameter of the outer needle was 1.10 mm, It was attached to syringe pump 1 filled with PAN solution as the external phase (shell materials), the feed speed was 0.4–0.6 mL·h^−1^. The diameter of the inner needle was 0.40 mm, which was attached to syringe pump 2 filled with healing agents as the internal phase (core materials), the feed speed was 0.04–0.06 mL·h^−1^. PAN solution (9 wt%) was prepared with PAN powders dissolved in DMF by magnetic stirring at ambient temperature for 24 h. The healing agent was prepared with EPON826 mixed with photoinitiator PI 6992 (10:1, w/w) and then ultrasonicated for 30 min. Positive (coaxial needle) and negative (aluminum substrate) voltages were 11 and −2 kV, respectively. For nanofibers collection, the needle was perpendicular to the aluminum substrate (75 × 15 × 0.5 mm), and the distance between them was 20 cm. After 30 min of collection, the nanofibers were dried at 60 °C for 8 h. In a contrast, pure PAN solid nanofibers without any core materials were prepared under the same conditions as a normal single‐needle.

### Preparation of Composite Coatings

A needle with a diameter of 0.52 mm was connected to the syringe pump filled with a mixture of EPON862 and PA (1:1, w/w). The needle was perpendicular to the aluminum substrate with a distance of 15–20 cm. The injection speed of a single syringe pump was 0.9 mL·h^−1^, and positive (needle) and negative (aluminum substrate) voltages were 11 and −0.5 kV, respectively. The mixture was deposited on the aluminum substrate with core–shell nanofibers on the surface by the electro‐microfluidic spinning instrument until the nanofibers were covered completely. In contrast, the mixture was deposited on the aluminum substrate covered with pure PAN solid nanofibers and on the aluminum substrate without any fibers, respectively. After 30 min of deposition, the whole was cured at ambient temperature for 24 h, and composite coatings containing core–shell nanofibers (abbreviated as composite coatings), composite coatings containing pure PAN solid nanofibers (abbreviated as PAN@coatings), and coatings without any fibers (abbreviated as pure resin coatings) were obtained.

## Conflict of Interest

The authors declare no conflict of interest.

## Supporting information



Supporting Information

Supplemental Video 1

Supplemental Video 2

## Data Availability

The data that support the findings of this study are available from the corresponding author upon reasonable request.

## References

[1] L. Jiang , Y. Dong , Y. Yuan , X. Zhou , Y. Liu , X. Meng , Chem. Eng. J. 2022, 430, 132823.

[2] L. Pengpeng , F. Xue , L. Xin , X. Li , Y. Fan , J. Zhao , L. Tian , J. Sun , L. Ren , ACS Appl. Mater. Interfaces 2023, 15, 7538. 36706036 10.1021/acsami.2c19132

[3] Y. Wang , L. Zhao , Y. Zhao , W. Y. Wang , Y. Liu , C. Gu , J. Li , G. Zhang , T. J. Huang , S. Yang , Adv. Mater. 2018, 30, 1870395. 10.1002/adma.201805686 30311312

[4] R. Davidson , A. Verma , D. Santos , F. Hao , C. Fincher , S. Xiang , J. Van Buskirk , K. Xie , M. Pharr , P. P. Mukherjee , S. Banerjee , ACS Energy Lett. 2019, 4, 375.

[5] J. Wang , B. Chen , H. Lin , R. Li , Sci. Total Environ. 2023, 881, 163135. 37003320 10.1016/j.scitotenv.2023.163135

[6] P. Xiong , J. Yan , P. Wang , Z. Jia , W. Zhou , W. Yuan , Y. Li , Y. Liu , Y. Cheng , D. Chen , Y. Zheng , Acta Biomater. 2019, 98, 160. 31029829 10.1016/j.actbio.2019.04.045

[7] Y. Wu , W. Zhao , J. Ou , Adv. Colloid Interface Sci. 2021, 295, 102494. 34343903 10.1016/j.cis.2021.102494

[8] S. H. Cho , S. R. White , P. V. Braun , Adv. Mater. 2009, 21, 645.

[9] H. Li , L. Xin , J. Gao , Y. Shao , Z. Zhang , L. Ren , Small 2024, 20, 2309012. 10.1002/smll.202309012 38178643

[10] R. Li , S. Tian , Y. Tian , J. Wang , S. Xu , K. Yang , J. Yang , L. Zhang , Small 2023, 19, 2206075. 10.1002/smll.202206075 36534911

[11] J. Shi , L. Xu , D. Qiu , Adv. Sci. 2022, 9, 2200072. 10.1002/advs.202200072 PMC9109053 35285176

[12] N. Horiuchi , Nat. Photonics 2023, 17, 136.

[13] A. Habibiyan , B. Ramezanzadeh , M. Mahdavian , G. Bahlakeh , M. Kasaeian , Chem. Eng. J. 2020, 391, 123630.

[14] R. Mohammadkhani , M. Ramezanzadeh , S. Saadatmandi , B. Ramezanzadeh , Chem. Eng. J. 2020, 382, 122819.

[15] Y. Ye , D. Yang , D. Zhang , H. Chen , H. Zhao , X. Li , L. Wang , Chem. Eng. J. 2020, 383, 123160.

[16] X. Sun , S. Gu , L. Wang , H. Wang , S. Xiong , X. Yin , S. Yang , J. Colloid Interface Sci. 2024, 654, 25. 37832232 10.1016/j.jcis.2023.09.182

[17] L. Winkless , Mater. Today 2018, 21, 929.

[18] W. Wang , L. Cao , Q. Li , C. Du , S. Chen , J. Colloid Interface Sci. 2023, 630, 511. 36334487 10.1016/j.jcis.2022.10.089

[19] T. Yimyai , D. Crespy , M. Rohwerder , Adv. Mater. 2023, 2300101. 10.1002/adma.202300101 36939547

[20] L. Ma , J. Wang , D. Zhang , Y. Huang , L. Huang , P. Wang , H. Qian , X. Li , H. A. Terryn , J. M. C. Mol , Chem. Eng. J. 2021, 404, 127118.

[21] Y. B. Chong , D. Sun , X. Zhang , C. Y. Yue , J. Yang , Chem. Eng. J. 2019, 372, 496.

[22] Y. Feng , Y. Cui , M. Zhang , M. Li , H. Li , Macromol. Mater. Eng. 2021, 306, 2000581.

[23] Y. Yu , J. Guo , H. Zhang , X. Wang , C. Yang , Y. Zhao , The Innovation 2022, 3, 100209. 35199079 10.1016/j.xinn.2022.100209 PMC8842082

[24] R. Wang , L. Cao , W. Wang , Z. Mao , D. Han , Y. Pei , Y. Chen , W. Fan , W. Li , S. Chen , ACS Appl. Mater. Interfaces 2024, 16, 42748. 39082737 10.1021/acsami.4c09260

[25] W. Zheng , Z. Li , C. Zhang , B. Wang , Q. Zhang , Q. Wan , L. Kong , L. Li , Nano Res. 2019, 12, 1461.

[26] X. Lu , Y. Hu , J. Guo , C. Wang , S. Chen , Adv. Sci. 2019, 6, 1901694. 10.1002/advs.201901694 PMC6864515 31763152

[27] K. Zhang , W. Zhao , Q. Liu , M. Yu , Sci. Rep. 2021, 11, 8895. 33903691 10.1038/s41598-021-88520-0 PMC8076242

[28] R. P. L. Zárate , T. N. Veras , J. M. Nascimento , I. M. G. Santos , E. S. Medeiros , J. Appl. Polym. Sci. 2024, 141, e56011.

[29] X. Ji , W. Wang , X. Zhao , L. Wang , F. Ma , Y. Wang , J. Duan , B. Hou , J. Mater. Sci. Technol. 2022, 101, 128.

[30] L. Cao , W. Wang , J. Cheng , T. Wang , Y. Zhang , L. Wang , W. Li , S. Chen , ACS Appl. Mater. Interfaces 2023, 15, 48645. 37791906 10.1021/acsami.3c10698

[31] L. Cao , Q. Wang , W. Wang , Q. Li , S. Chen , ACS Appl. Mater. Interfaces 2022, 14, 27168. 10.1021/acsami.2c05048 35666307

[32] Y. Song , X. Yu , S. Chen , J. Polym. Sci. 2024, 62, 447.

[33] X. Fu , W. Du , H. Dou , Y. Fan , J. Xu , L. Tian , J. Zhao , L. Ren , ACS Appl. Mater. Interfaces 2021, 13, 57880. 34797646 10.1021/acsami.1c16052

[34] S. Shen , H. Zhang , K. Song , Z. Wang , T. Shang , A. Gao , Q. Zhang , L. Gu , W. Zhong , Angew. Chem., Int. Ed. 2024, 63, e202315340. 10.1002/anie.202315340 37985934

[35] X. Wang , C. Jing , Y. Chen , X. Wang , G. Zhao , X. Zhang , L. Wu , X. Liu , B. Dong , Y. Zhang , J. Magnes. Alloys 2020, 8, 291.

[36] Y. Zhou , Y. Ma , Y. Sun , Z. Xiong , C. Qi , Y. Zhang , Y. Liu , ACS Appl. Mater. Interfaces 2019, 11, 6512. 30668101 10.1021/acsami.8b19663

[37] Standard A. D265 , Standard Test Methods for Determining the Izod Pendulum Impact Resistance of Plastics, ASTM International, 2010.

[38] H. Wang , H. Cai , B. Chen , C. Mao , Polym. Compos. 2021, 42, 3281.

[39] Y. Dzenis , Science 2008, 319, 419. 18218884 10.1126/science.1151434

[40] A. Cohades , C. Branfoot , S. Rae , I. Bond , V. Michaud , Adv. Mater. Interfaces 2018, 5, 1800177.

[41] Q. Xu , Y. Si , R. He , X. Qi , X. Su , Y. Fu , ACS Appl. Mater. Interfaces 2022, 14, 6005. 35050593 10.1021/acsami.1c21874

