# Supplementary material for: Microfluidic Electrospinning Core–Shell Nanofibers for Anti‐Corrosion Coatings With Efficient Self‐Healing Properties
Source: Adv Sci (Weinh). 2024 Dec 18;12(6):2409751. doi: 10.1002/advs.202409751 (PMC11809378; doi:10.1002/advs.202409751)
Supplement: Supplementary file 1 — Supporting Information [file ADVS-12-2409751-s001.docx]

**ID:** No. advs.202409751R1

**Article type:** Research Article

Supporting Information

**Microfluidic Electrospinning Core-Shell Nanofibers for** **Anti-Corrosion Coatings with Efficient Self-Healing Properties**

*Qingqing Tang^1^, Cuiping Ji^1^*, Guoying Wei^1^*, Jing Hu^1^, Feifan Chang^1^, Benfeng Zhu^1^, Li Ren^1^, Dongliang Peng^1,2^*

^1^College of Materials and Chemistry, China Jiliang University, Hangzhou 310018，P. R. China

^2^Department of Materials Science and Engineering, College of Materials, Xiamen University, Xiamen 361005, P. R. China

*****Corresponding Authors**

E-mail address：cuipingji@cjlu.edu.cn (C. Ji), guoyingwei@cjlu.edu.cn (G. Wei)

**
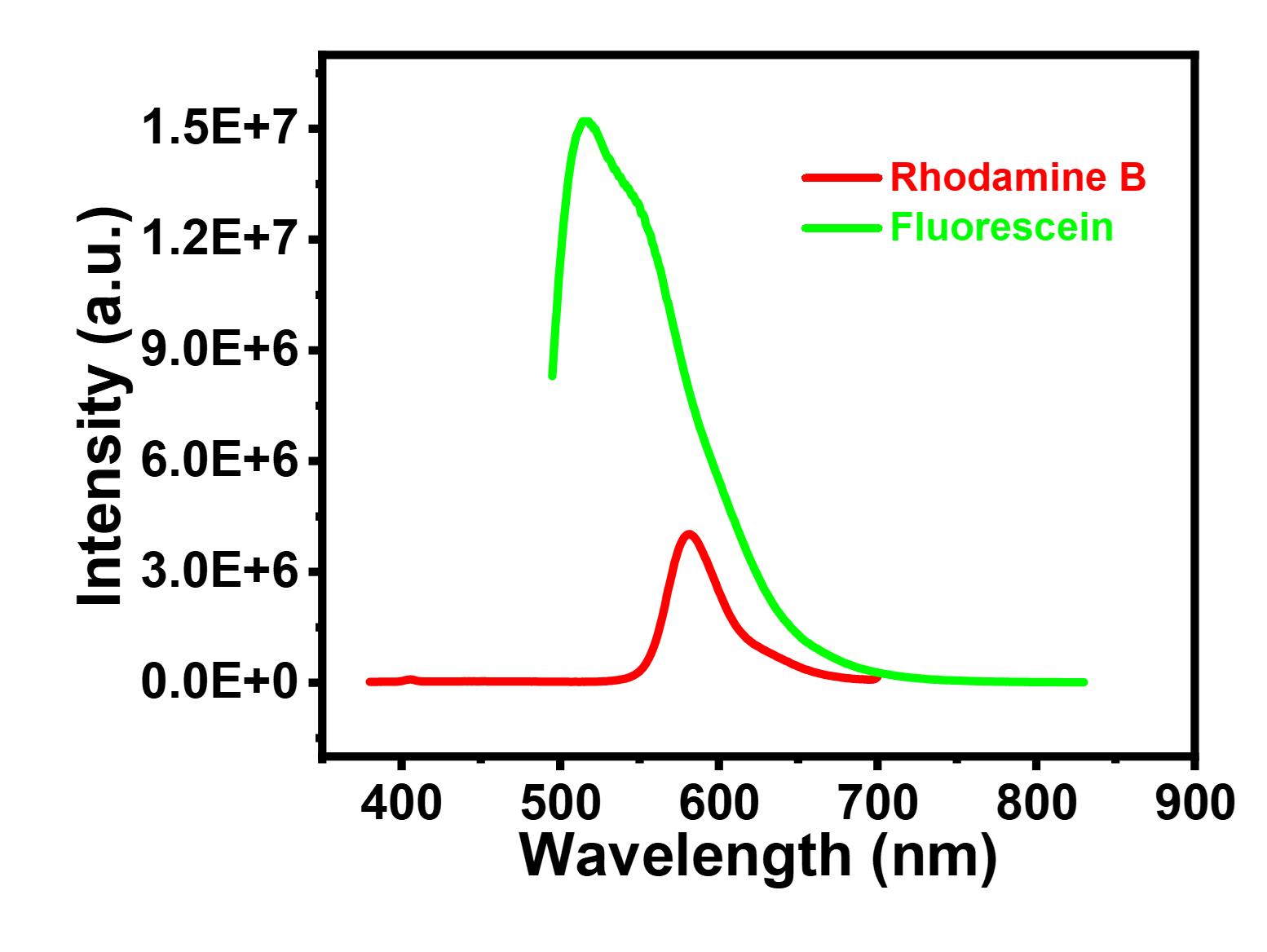
**

**Figure S1.** Fluorescence emission spectra of rhodamine B and fluorescein in aqueous solution.


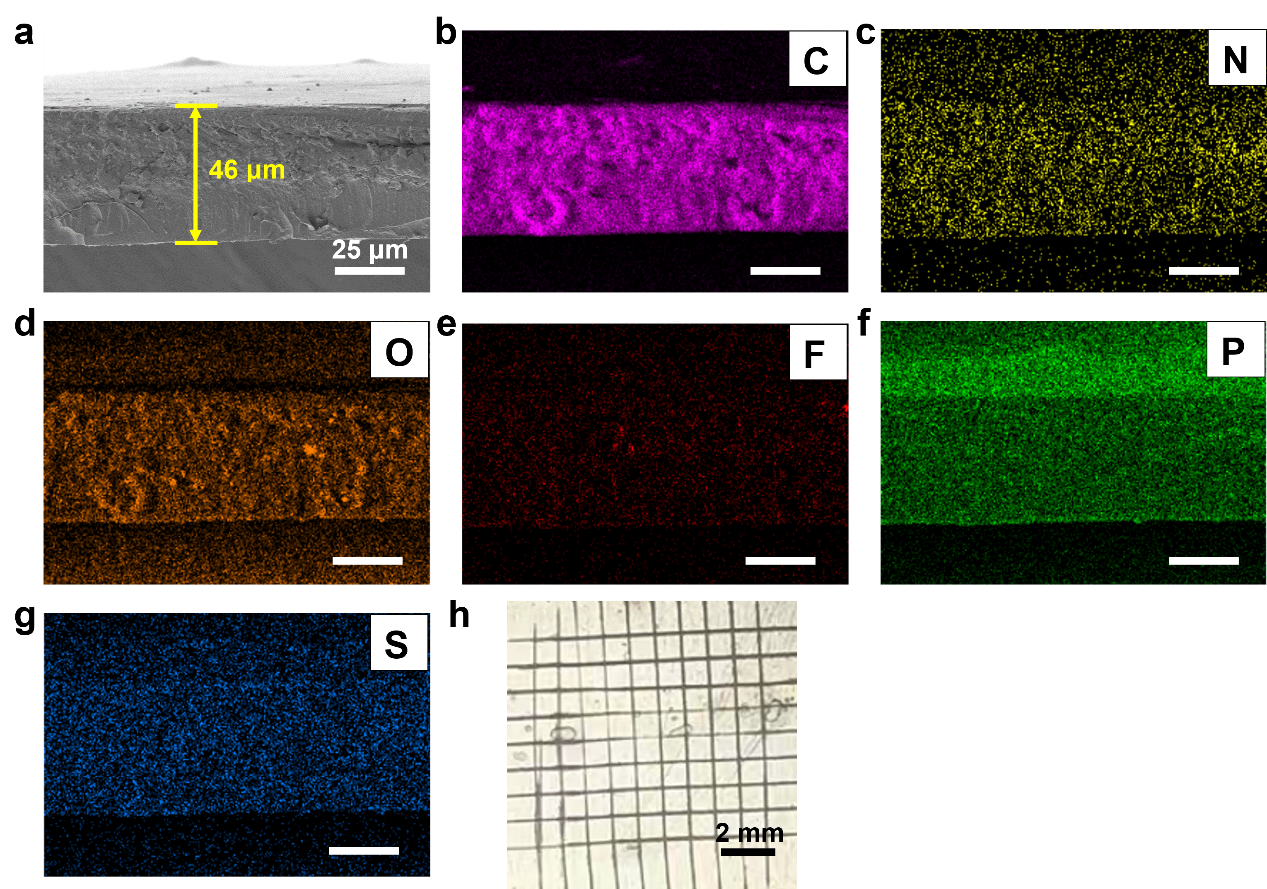


**Figure S2.** a) The SEM image of the coating cross-section view and the EDS mapping results of b) C, c) N, d) O, e) F, f) P, g) S. h) Composite coatings after the tape-peeling test.


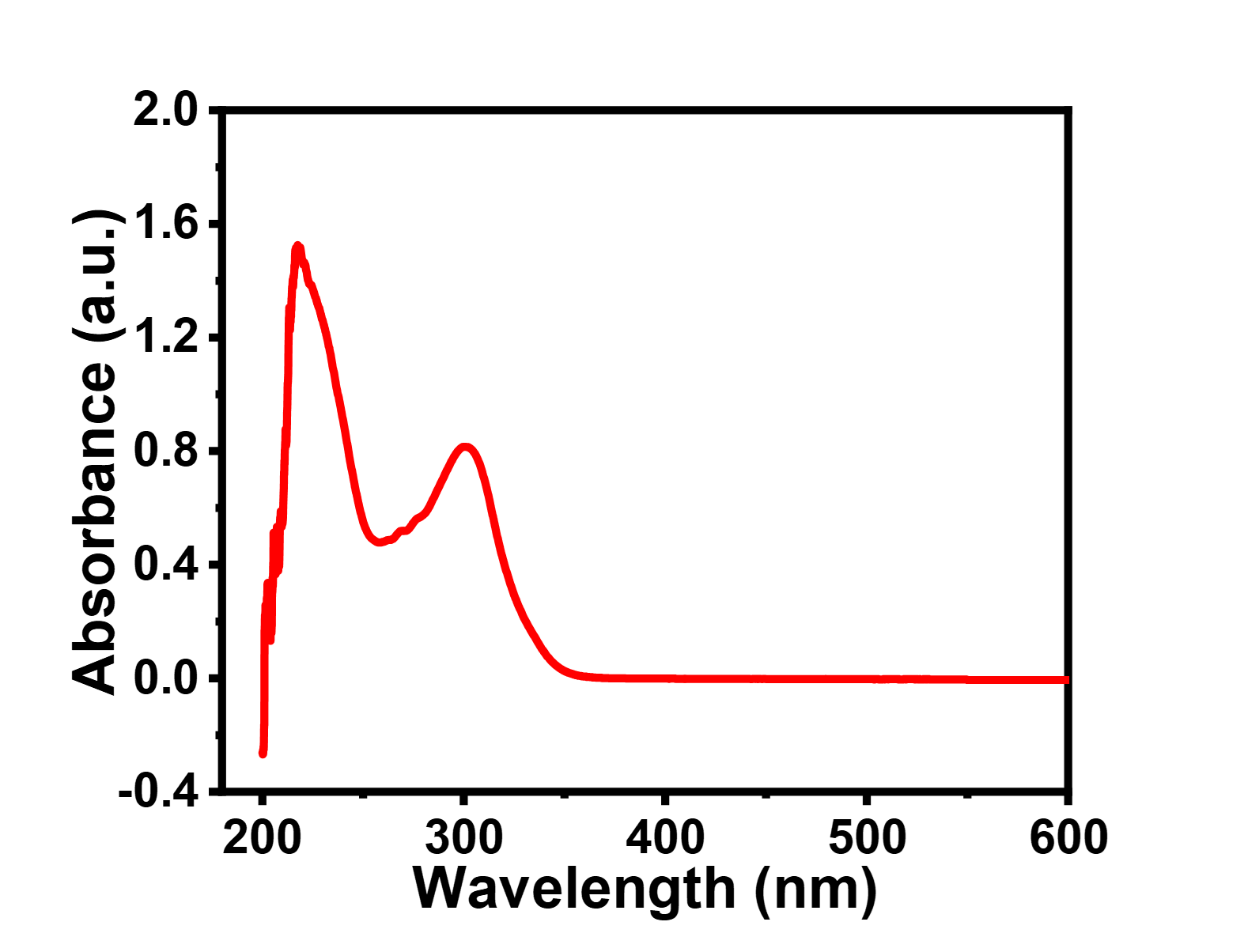


**Figure S3.** UV-vis absorption spectrum of cationic photoinitiators (PI 6992) in propylene carbonate solution.

**
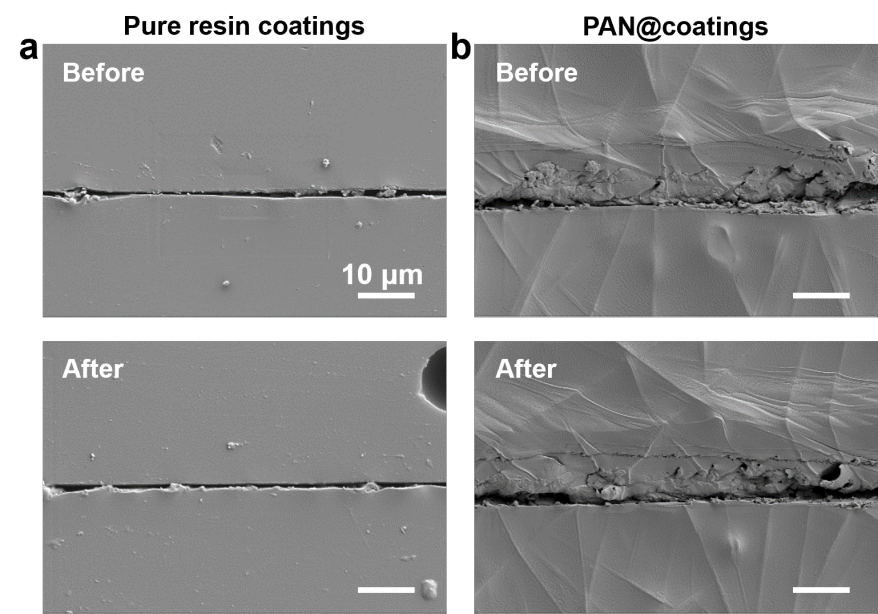
**

**Figure S4.** SEM images of a) pure resin coatings and b) PAN@coatings with a crack before and after UV radiation for 30 min.


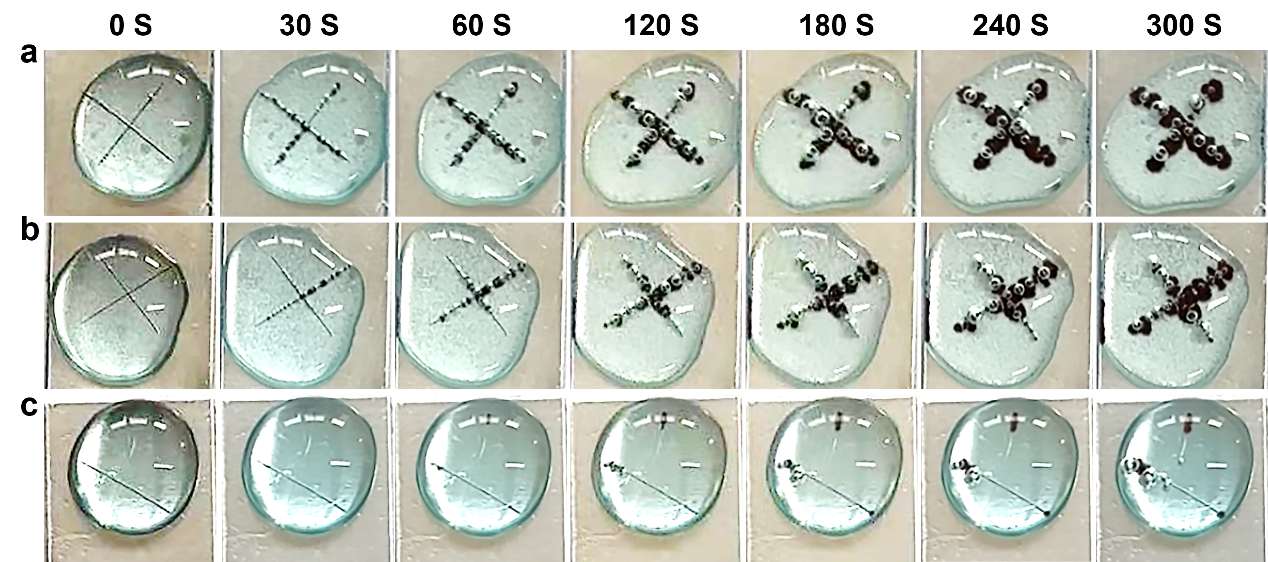


**Figure S5.** Photographs of a) pure resin coatings, b) PAN@coatings and c) composite coatings after dropped copper sulfate solution for 0 s, 30 s, 60 s, 120 s, 180 s, 240 s, 300 s.


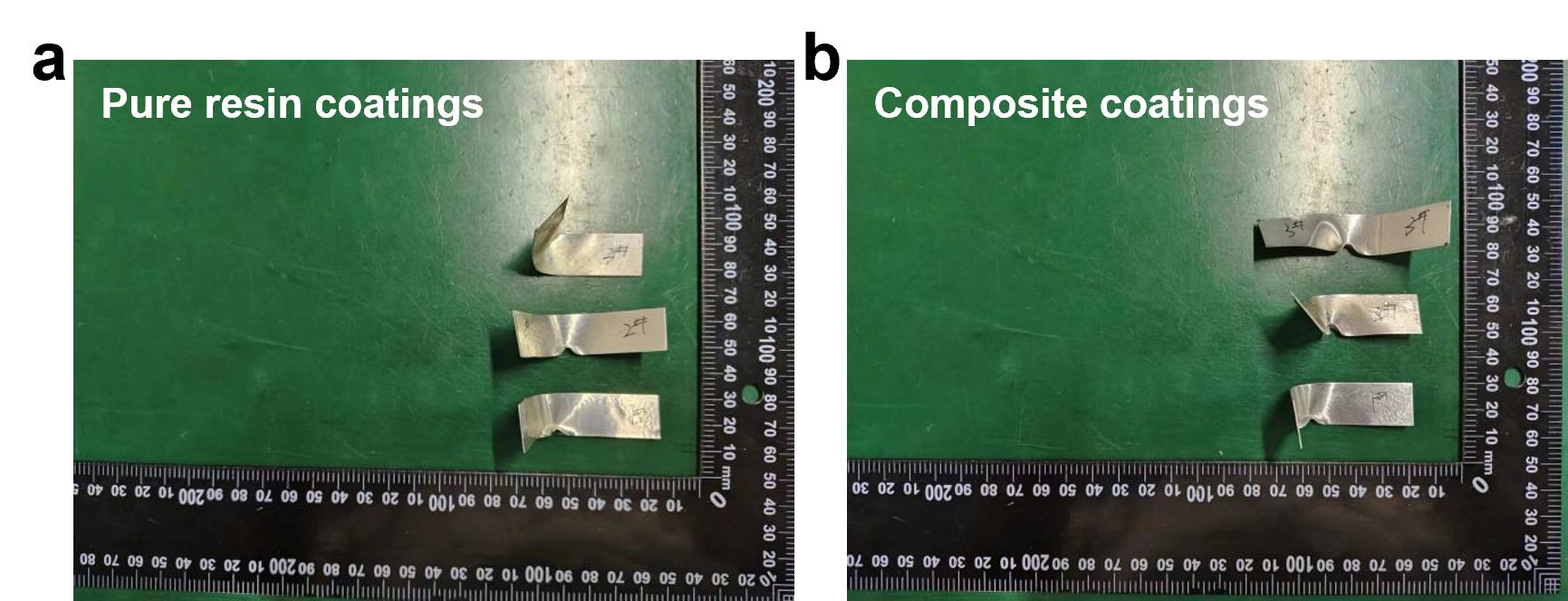


**Figure S6.** Photographs of a) pure resin coatings and b) composite coatings after the charpy pendulum impact test.

**Table S1.** XPS fitting results of Al 2p atomic concentration.

| Samples | Concentration (%) |
| --- | --- |
| Pure resin coatings | 9.33 |
| PAN@coatings | 4.79 |
| Composite coatings | 3.26 |

**Table S2.** Mechanical properties of pure resin coatings and composite coatings.

| Samples | Tensile modulus  (MPa) | Tensile strength  (MPa) | Elongation at break  (%) | Bending strength  (MPa) | Impact toughness  (J·cm^-2^) |
| --- | --- | --- | --- | --- | --- |
| Pure resin coatings | 19238.86 ± 1237.25 | 162.83 ± 7.69 | 23.94 ± 3.09 | 196.95 ± 13.11 | 15.93 ± 2.86 |
| Composite coatings | 21070.85 ± 2776.05 | 182.90 ± 15.32 | 29.61 ± 1.17 | 250.97 ± 28.80 | 16.77 ± 2.64 |
